# Smoking Status and Well-Being of Underserved African American Older Adults

**DOI:** 10.3390/bs10040078

**Published:** 2020-04-15

**Authors:** Mohsen Bazargan, Sharon Cobb, Jessica Castro Sandoval, Shervin Assari

**Affiliations:** 1 Departments of Family Medicine, College of Medicine, Charles R Drew University of Medicine and Science, Los Angeles, CA 90059, USA; mohsenbazargan@cdrewu.edu; 2 Departments of Family Medicine, University of California, Los Angeles (UCLA), Los Angeles, CA 90095, USA; 3 School of Nursing, Charles R Drew University of Medicine and Science, Los Angeles, CA 90059, USA; sharoncobb@cdrewu.edu; 4 Department of Public Health, Charles R Drew University of Medicine and Science, Los Angeles, CA 90059, USA; jessicacastrosandoval@cdrewu.edu

**Keywords:** smoking, African Americans, older adults, well-being, depression, self-rated health, quality of life

## Abstract

*Purpose*: This study investigated the association between current and past cigarette smoking, with four domains of well-being, namely, physical quality of life, mental quality of life, depressive symptoms, and self-rated health status (SRH), among older African American adults who lived in economically impoverished areas of South Los Angles. *Methods*: This community-based cross-sectional study included a convenience sample of economically-disadvantaged African Americans adults (n = 740) who were 55 years old and older residing in South Los Angeles. We conducted in-depth face-to-face interviews to collect data on the socioeconomic status (level of education and fiscal pressures), demographic factors (age and gender), physical health (number of chronic medical conditions), smoking (never smokers (reference group), past smokers, and current smokers), and well-being (quality of life, depressive symptoms, and self-rated health). Linear regressions were used to analyze the data. *Results*: Over 21% reported that they are current smokers, compared with 31% who identified as former smokers. Almost 40% perceived the quality of their health status to be fair or poor. Compared with non-smokers, current cigarette smokers reported a worse physical quality of life, depressive symptoms, and self-rated health. Current smokers also reported a marginally worse mental quality of life. Past smoker status was inconsistently associated with worse well-being in some, but not all, indicators. The association between smoking status and worse well-being was independent of gender, socioeconomic status, and physical health status. *Conclusion*: Current smoking is associated with worse well-being of older African American adults in economically constrained urban settings. As the same pattern could not be found for former smokers, quitting smoking may be a strategy for economically-disadvantaged African American individuals to enhance their well-being. This provides additional support for programs that help African American individuals who are smokers to quit smoking, particularly in economically-disadvantaged urban areas.

## 1. Background

Socio-economic status (SES) is a significant predictor of use of tobacco products, with tobacco use being more common in low SES groups of society [1]. According to the United States National Health Interview Survey (NHIS), the rate of smoking among individuals with an annual income of less than $35,000 is almost three times higher than those with an annual income of $100,000 or more (7.3 vs. 21.3) [1].

Even though there is a well-documented association between smoking and health status and demographic factors, the degree of this relationship is not clear. Even though it has been linked to many chronic diseases, causal pathways linking smoking behaviors and social factors may be harder to determine. However, we know that smoking is positively associated with psychological distress [1], depressive symptoms [2,3,4,5,6], low quality of life [7,8,9,10,11], and poor self-rated health [12,13,14]. However, it is unclear how smoking is influencing the quality of life of underserved minority populations with low SES. Very few studies have examined the correlations and consequences of the use of tobacco products among underserved and vulnerable populations, specifically, underserved populations of color with low SES. The smoking status of underserved and under-resourced older African American adults with co-morbidities requires additional research attention.

The NHIS data show that non-Hispanic Whites and African Americans have similar rates of smoking (15.0% vs. 14.6%) [1]. However, this data suggest that African Americans and non-Hispanic Whites have different patterns of smoking initiation and cessation. While African Americans have a lower probability of initiation of smoking at a young age, they also have a lower probability of cessation of smoking in middle and older age than their non-Hispanic White counterparts. The different patterns of initiation and cessation [15,16,17] give rise to distinct patterns of epidemiology. Rates of tobacco use among African Americans are very similar to non-Hispanic Whites, even though initiation is lower and later for African Americans [18].

Even though NHIS data show that non-Hispanic Whites and African Americans have similar rates of smoking, African Americans carry an increasing burden of tobacco-related disease [19,20,21,22]. This is probably due to an increased vulnerability of African Americans to undesired outcomes, because of the telescoping effect [23]. Local community-based data show a much higher rate of smoking among underserved older African American adults [4]. The high prevalence of smoking among older African American adults is significant, because many of the health conditions of smoking tend to occur later in life [18]. In addition, older African American adults are more likely than their White counterparts to suffer from major chronic conditions, which may exacerbate the health risks of smoking for this population. For example, older African American adults tend to have higher rates of cardiometabolic diseases [24], diabetes [25], and lung disease [26] when compared with their white counterparts. According to the data from the Centers for Medicare and Medicaid Services (CMS), 71% of non-Hispanic African American older adults have hypertension, 41% have diabetes, and 19% have heart failure [27]. The comparable figures for older non-Hispanic Whites adults were hypertension (60%), diabetes (25%), and heart failure (15%). Similarly, the prevalence of asthma is 5.9% and 4.5% among African American and non-Hispanic White older adults, respectively [27].

## 2. Aim

Therefore, our study aims to examine the association between smoking status and various domains of well-being of middle-aged and older African American adults. Thus, we compare the physical and mental quality of life, depressive symptoms, and perceive health (self-rated health) across current-, past-, and never-smokers in a large community-based sample of underserved middle-aged and older African American adults. Such information will enable healthcare providers to have a better understanding of the issues surrounding the use of tobacco products among older African American adults. Although very limited studies on smoking in underserved older African American adults are emerging, further empirical research on older African American smokers could inform the development of interventions tailored to this segment of the population. Continued surveillance of smoking at the community level is critical for informing smoking cessation efforts for this at-risk population.

## 3. Methods

This community-based cross-sectional study surveyed a convenience sample (n = 740) of older African Americans residing in South Los Angeles [3,4,6,28,29,30,31]. Also referred to as “South Los Angeles” Service Planning Area 6 of Los Angeles County, which serves the communities of Athens, Compton, Crenshaw, Florence, Hyde Park, Lynwood, Paramount, and Watts. Together, these areas comprise a population of over one million. South Los Angeles is one of the most underserved and under-resourced service planning areas in the County of Los Angeles. Close to 96% of the population of SPA 6 are Latino and African Americans. Nearly 34% of the residents of SPA 6 have household incomes below the federal poverty guidelines. One out of four adults in SPA 6 has been diagnosed with hypertension and age-adjusted coronary heart disease. The age-adjusted diabetes death rate per 100,000 population in SPA 6 is 37.6 compared with 7.5 in the West Los Angeles Service Planning Area (SPA 5).

Eligible participants for this study include older, non-institutionalized African American adults aged 55 years or older. Within the older African American adult population, almost 37% are between the ages of 50 and 64, and 48% are 65 years of age and older. This subset of older adults is faced with numerous health disparities, including lower rates of preventative health services and a greater delay in care. Individuals enrolled in any type of nursing facility, enrolled in any other research study, and who have observable intellectual impairments, were excluded from this study [3,4,6,28,29,30,31].

*Recruitment:* Our research team located several areas in South Los Angeles largely populated or visited by our target population. These areas include community areas, such as senior centers, residential apartments, and African American churches, located in SPA 6. Potential participants were recruited through various methods, including flyers in community areas and healthcare clinics. In addition, potential participants were referred by healthcare providers and already-enrolled participants [3,4,6,28,29,30,31].

*Ethical Aspects:* The study protocol was authorized by the Charles R. Drew University of Medicine and Science (CDU IRB #: 14-12-2450-05) institutional review board (IRB). Before being enrolled in this study, all participants were screened for eligibility and signed written informed consent. Structured face-to-face interviews were conducted in the homes of participants so as to collect study data. Following the completion of the study, participants received financial compensation for providing their time.

## 4. Measurement

### 4.1. Demographic and SES Characteristics

The demographic covariates examined were age and gender. Age was classified as an interval variable, and gender was classified as a binary variable (coded as 1 = female, 0 = male). The SES measures of focus in this study were the level of education and fiscal pressures, which were operationalized as interval variables. SES is strongly correlated to education; accordingly, when measuring the level of education, we looked at self-reported years of schooling, where a lower score indicated a low SES, and a higher score indicated a higher SES. Pearlin’s list of continuous financial adversities experienced by low SES individuals [32] was the basis for the items used to measure fiscal pressures. The items measured incorporate fiscal pressures, such as the inability to cover basic necessities like food or bills, because of a lack of funds or low funds, using a Likert-type scale (“never” to “always”). The fiscal pressures were calculated (Cronbach’s alpha = 0.923), with a higher score indicating lower SES.

### 4.2. Cigarette Smoking Behaviors

Participants responded to a single item question about describing their cigarette use, where the responses given were “never smoked”, “previously smoked”, and “current smoker”.

### 4.3. Comorbid Medical Conditions (CMCs)

The CMCs measured were characterized by the number of comorbid conditions. Participants were asked if their medical provider had ever confirmed if they had any of the following medical ailments: hypertension, back pain, diabetes, thyroid disorder, cancer, heart disease, arthritis, heart burn, migraine, or stroke. The use of self-reported data is a credible measure to compile information on comorbid medical conditions [33,34,35,36]. Nevertheless, there is skepticism about the ability of this method to measure comorbid conditions.

### 4.4. Depressive Symptoms

The Short Form Geriatric Depression Scale was used to measure depressive symptoms and to determine level of depression. The scale consists of 15 items with a “no” or “yes” response, and generates a summary score that ranges from 0 to 15 [37,38,39,40]. The summary score ranges indicate no depression, mild depression, moderate depression, and severe depression; thus, a higher score indicates more depression. This assessment tool has been used with confidence in community and health care settings for its first-rate reliability and validity in measuring depressive symptoms among the geriatric population [37,38,39,40].

### 4.5. Physical and Mental Health-Related Quality of Life (PCS and MCS)

Quality of life was measured using SF-12v2, a 12-item health assessment tool focusing on physical and mental health dimensions. The items in this assessment tool are based on the following eight sub-domains (or subscales): bodily pain (BP), general health (GH), vitality (VT), and social functioning (SF) (each with one item); and physical functioning (PF), mental health (MH), role physical (RP), and role emotional (RE) domains (each with two items). This assessment tool generates two summary scores correlating to the Physical Component Summary (PCS) and the Mental Component Summary (MCS) scores. The summary scores were calculated from the z-scores of the eight subscales. The scoring method used was guided by the methods proposed by the original authors. All of the subscales contribute to the scoring of the two dimensions in different proportions, using the weights from the principal component analysis from the original 36-item scale. The SF-12v12 scale uses country-specific coefficient scoring, where population scores have a mean of 50 and a standard deviation of 10 for the United States population. Summary scores can be compared to this standard, where a higher score is demonstrative of better health related to quality of life [41,42,43,44].

*Self-Rated Health:* Study participants’ overall health was measured by a single item, where participants provided responses between 1, indicating “excellent”, and 5, indicating “poor”. Poorly perceived health is a strong predictor of early deaths in a population [45,46], regardless of cause in clinical and community settings, and is used in this study to capture unrecognized symptoms.

## 5. Data Analysis

We conducted the data analysis using SPSS 23.0. The characteristics of our sample were described using the mean, standard deviations (SD), frequency (n), and relative frequency (%). For our bivariate analysis, we used Analysis of Variance (ANOVA) to compare current smokers, past smokers, and non-smokers. We reported the mean, standard deviation of each group, and a *p* value for the comparison of the three groups. For our multivariable model, linear regression models were used. In these models, depressive symptoms, self-rated health status, and PCS and MCS dimensions for quality of life were the dependent variables; smoking status, as two dummy variables, were the independent variables; and age, gender, education, financial strain, and chronic medical conditions, were the confounders. This approach enabled us to estimate the independent impacts of current smoking and ex-smoking on our outcome variables. We reported regression coefficients (b), standard error (SE), confidence intervals (95% CI), and *p* values.

## 6. Results

### 6.1. Descriptive Statistics

Table 1 summarizes the described characteristics of the variables examined in the sample. Study participants were age 55 and older, with the average age being 71.7 years old. The mean number of years of schooling (education) was 12.7 years.

Participants were mostly females (64.1%). One out of four participants indicated that they had no formal education beyond eleventh grade. Slightly less than 36% reported completing high school. With regard to health status, only 6.2% of the sample rated their present health as excellent; 36% described their health as good, and 31% and 7% rated their health as fair and poor, respectively. More than 30% and 35% reported that they have been diagnosed with heart conditions and with diabetes mellitus, respectively. More than 21% (160) of participant self-identified as current regular smokers, whereas 31% (231) admitted that they had stopped smoking. However, 47% (347) reported that they never smoked cigarettes.

### 6.2. Bivariate Correlations

Table 2 also compares the study variables between current-, past-, and never-smokers. Compared with never- and past-smokers, current-smokers were younger, reported more financial strains, a higher number of comorbid medical conditions, and depressive symptoms. In addition, current-smokers reported lower levels of physical and mental health-related quality of life (PCS and MCS, respectively).

### 6.3. Multivariate Analysis

Table 3 reports the result of four different multiple linear regressions, one for each outcome. In these models, two smoking indicators were the predictors, and SES and health status were the confounders. Current smoking was associated with worse depressive symptoms (b = 0.674, beta = 0.100, *p* = 0.009), self-rated health (b = 241, beta = 0.098, *p* = 0.019), and physical quality of life (b = −3.65, beta = −0.124, *p* = 0.001). Current smoking also showed a marginal association with a poor mental quality of life (b = −1.94, beta = −0.073, *p* = 0.078). Past smoking was associated with poor physical quality of life (b = −2.03, beta = −0.077, *p* = 0.028), but with none of the other outcomes.

The number of chronic medical conditions was correlated with all of the outcomes. Financial strain was another universal determinant of physical and mental quality of life, depressive symptoms, and self-rated health status. However, education failed to predict any of the outcomes. Gender was associated with all of the outcomes, with physical quality of life being the only exception. Age was only associated with physical quality of life, but not mental quality of life, depressive symptoms, or self-rated health status.

## 7. Discussion

Compared with non-smokers, current cigarette smokers reported a worse physical quality of life, more depressive symptoms, and worse self-rated health. Similarly, current smokers also reported a marginally worse mental quality of life. Past smoker status, however, was only associated with a worse physical quality of life. As the same pattern could not be found for former smokers, quitting smoking may be a strategy for older, economically-disadvantaged African American adults to enhance their well-being. This provides additional support for programs that help African American individuals quit smoking, particularly those in economically-disadvantaged urban areas.

Poor mental quality of life was marginally associated with current smoking status. Recent literature has shown that current smokers have a worse SF-12 mental component summary score, indicating a poor mental quality of life [47,48]. Similar epidemiological studies on global populations uncovered that tobacco smokers reported a poorer quality of life [49]. Schmitz and colleagues (2003) found that over half of almost 1200 smokers had at least one mental illness [49]. Moreover, mental illness is associated with heavier smoking among African Americans. This group of adult smokers are also at risk for cognitive impairment [50], indicating that their mental quality of life may be severely compromised as a result of habitual smoking.

Our findings revealed that current smoking status is associated with a worse physical quality of life using the SF-12. Related literature has demonstrated that current smokers with a chronic illness have significantly worse SF-12 physical quality of life scores [47,51]. Older current smokers are less likely to engage in physical activity, which may be a contributor to their lower physical quality of life [52]. Among African Americans, smoking is associated with multiple chronic conditions, such as kidney disease [53] and respiratory illnesses [54]. Managing multiple chronic conditions and negative health behaviors, such as smoking, may lead to poorer self-rated health in this population.

It is not surprising that current smoking is associated with overall well-being and quality of life. Smoking has been associated with both poor and fair self-rated health status in various groups [12,14]. Previous literature has documented that smoking has a negative association with various measures of quality of life [8], including oral health [7], and a lower self-rated quality of life in the general population [9]. Viana and colleagues (2019) assessed quality of life among older adult smokers using the World Health Organization Quality of Life and Quality of Assessment for Older Adults. Their findings revealed that those with a higher tobacco dependence reported higher levels of fear and concern about death [55]. Among older, low-income African American adults, the probability of smoking frequency has been shown to increase with greater perceived stress [21]. Further research should assess smoking characteristics among older African Americans, with widely used quality of life measures.

Depressive symptoms were revealed to be associated with current smoking use, which is supported by previous studies [47,56]. A population-based study in Belgium found that smoking use was associated with depressive symptoms [11]. Smokers have a 43% greater risk of developing depressive symptoms than non-smokers [50]. A critical review of the literature uncovered that depressed adults are more likely to be dependent on nicotine, less likely to quit smoking, and more likely to relapse [57]. Among African Americans, individuals with higher levels of depression were found to be more likely to smoke [58]. The current study adds to the growing literature examining the relationship between depression and smoking in this population.

Patterns of smoking cessation vary among African Americans compared with Whites. Historically, African Americans have lower odds of smoking cessation compared with Whites [59], which may lead to increased years of smoking and increased adverse health effects [18]. Jones and colleagues found that African Americans smoked for an estimated two years longer before quitting when compared with Whites [60]. Minority groups, specifically African Americans, are more likely to quit smoking cigarettes within a 20-year span, compared with Whites [15], which may be due to worsening health status and decline. Compared with Whites, African American smokers continue to report higher levels of everyday discrimination, which is associated with tobacco dependence.

Previous literature that focused on smoking in older, economically-disadvantaged African Americans adults have found similar results. A recent study that analyzed this sample of older African American adults residing in South Los Angeles found that even though men smoked more than women, depressive symptoms had a stronger effect on women [4]. Current smoking use among this group is also associated with higher financial difficulties [2] and higher odds of drinking/binge drinking [3]. Smoking is a major modifiable risk factor for multiple illnesses, specifically, chronic respiratory conditions, among older African American adults living in these impoverished urban environments [54]. These studies signify the importance of understanding the health status among this group of vulnerable older smokers.

Moreover, Minorities’ Diminished Returns (MDRs) posits that there are smaller health benefits for African Americans compared with Whites. Assari and colleagues (2019) uncovered that highly educated African Americans are a higher risk for chronic obstructive pulmonary disease, a respiratory illness commonly associated with smoking, compared with matched Whites [26]. Additionally, household income, education, marital status, and age were found to be predictors for cigarette smoking among African Americans [61]. Kao and colleagues (2019) revealed that low income African American smokers with a history of cessation attempts have poorer health-related quality of life than current smokers.

The cessation of smoking may lead to multiple advantages for improved health and well-being, including psychological improvement [62]. A systematic review of 26 studies focused on smoking cessation and mental health revealed that quitting smoking led to decreased anxiety, stress, and depression, with an increased positive mood [63]. Smoking cessation is also associated with longitudinal improvement in physical quality of life [64,65] and a decrease in depressive symptomatology [66,67], and a decrease in other risky behaviors, such as alcohol consumption [68].

However, specific determinants, such as various socioeconomic factors and smoking behaviors, may be barriers to quitting smoking. Starting in adolescence, African Americans are at high risk of initiating and continuing smoking behaviors, a fact that can be attributed to aggressive marketing by tobacco companies [69]. Higher education may have a weaker protective effect from exposure to these tobacco advertisements for minority groups, such as African Americans, compared with non-minority groups [16]. African American intermittent smokers have a higher dependence, history of daily smoking, and smoke more cigarettes per day compared with Whites [70]. Within some minority populations, unhealthy behaviors, such as smoking and drinking, may act as coping behaviors for SES-related factors, such as financial difficulty [2].

There are established programs and interventions that have led to successful smoking cessation, aside from traditional pharmacological treatment, such as bupropion. Targeted strategies, specifically cognitive behavioral therapy, was successful in assisting African Americans to remain abstinent from smoking [71,72]. Other cognitive-based interventions, such as dispositional mindfulness, have assisted African Americans in quitting smoking and in increasing their social support [73]. Innovative smoking cessation interventions, such as narrative communication [74], should be implemented in this group, in addition to routine clinical treatment.

Cigarette use among African Americans not only contributes to poor well-being and health status, but increases the burden of tobacco-related diseases [19]. Future interventions should focus on successful culturally-tailored smoking cessation programs and strategies for older African Americans, including both pharmacological aids and specialized counseling [75]. Special attention should center on increasing the health literacy of these adults, with specific strategies for those with failed attempts at smoking cessation, and those reporting poor physical and mental health [76,77].

## 8. Limitations

This study does have a few limitations. Because of the cross-sectional design of this study, causal inferences cannot be made. In addition, relevant data from the medical charts and historical records of the participants were not collected, but self-reported data on the health and chronic conditions were assessed, including quality-of-life measures. Third, smoking status was assessed by self-report. Finally, smoking usage patterns, including years and frequency of cigarette smoking, were not measured. Despite these limitations, this study contributes greatly to our growing knowledge of smoking behaviors and well-being among older, economically-disadvantaged African American adults.

## 9. Conclusions

These findings provide a more complete understanding of the physical and psychosocial factors associated with smoking among older, economically-disadvantaged African Americans. African American smokers have a poorer quality of life and well-being, and suffer disproportionately from tobacco-related diseases. This study highlights the urgent need to provide more resources in the form of well-being support and cessation programs tailored to this population.

## Figures and Tables

**Table 1:** Characteristics of participants underserved African American adults (n = 740).

Characteristics	Mean ± SD
Age (years: 55–96) Education attainment (1–16) Financial strains (1–5) Self-Rated Health Status (1–5) Number of Chronic Conditions (1–12) Mental Quality of Life (SF-12) Physical Quality of Life (SF-12) Depression (GDS: 0–15)	71.7 ± 8.36 12.7 ± 2.24 4.16 ± 1.13 3.13 ± 1.02 4.24 ± 2.11 52.3 ± 10.9 40.3 ± 12.2 2.47 ± 2.77

**Table 2:** Bi-variate association between smoking status and quality of life, depressive symptoms and self-rated health status (n = 740).

	Never-Smokers	Past-Smokers	Current-Smokers	Sig.
	n (%) Mean (SD)	n (%) Mean (SD)	n (%) Mean (SD)	
Gender Male Female	94 (35) 253 (53)	86 (33) 145 (31)	85 (32) 75 (16)	<0.001
Age	74.17 ± 7.64	72.52 ± 8.36	65.40 ± 6.43	<0.001
Education	12.88 ± 2.04	12.48 ± 2.60	12.83 ± 2.08	0.095
Financial Strains	1.47 ± 0.86	2.03 ± 1.15	2.34 ± 1.33	<0.001
Chronic Conditions	3.77 ± 1.89	4.47 ± 1.94	4.09 ± 2.01	<0.001
Self-Rated Health	2.96 ± 0.97	3.16 ± 0.979	3.48 ± 1.09	<0.001
Depressive Symptoms	1.79 ± 2.42	2.71 ± 2.60	3.57 ± 3.30	<0.001
Physical Quality of Life	43.09 ± 11.50	38.47 ± 12.31	36.99 ± 12.28	<0.001
Mental Quality of Life	54.10 ± 9.57	52.18 ± 10.55	48.61 ± 13.05	<0.001

**Table 3:** Multiple linear regression between physical and mental quality of life, depressive symptoms, self-rated health status, and smoking behaviors (n = 740).

	Depressive Symptoms (GDS)			Self-Rated Health			Mental Quality of Life (SF-12)			Physical Quality of Life (SF-12)		
	B	Beta	Sig.	B	Beta	Sig.	B	Beta	Sig.	B	Beta	Sig.
(Constant)	2.75	-	0.023	3.58	-	<0.001	44.58	-	<0.001	56.87	-	<0.001
Gender	−0.039	−0.118	0.001	−0.017	−0.136	0.001	0.115	0.088	0.026	0.075	0.051	0.161
Age	0.022	0.004	0.909	0.054	0.025	0.476	1.48	0.065	0.067	−2.63	−0.103	0.002
Education	−0.049	−0.039	0.236	−0.010	−0.022	0.545	0.418	0.085	0.017	−0.215	−0.039	0.237
Financial strains	0.736	0.299	<0.001	0.124	0.137	<0.001	−2.14	−0.221	<0.001	−2.00	−0.185	<0.001
Chronic conditions	0.385	0.271	<0.001	0.121	0.233	<0.001	−1.01	−0.180	<0.001	−2.40	−0.385	<0.001
Current smokers	0.674	0.100	0.009	0.241	0.098	0.019	−1.94	−0.073	0.078	−3.65	−0.124	0.001
Past smokers	0.151	0.025	0.472	0.020	0.009	0.807	0.463	0.020	0.605	−2.03	−0.077	0.028
Adjusted *R*^2^	**0.272**			**0.140**			**0.148**			**0.279**		
